# Vegetation Dynamics and Recovery Potential in Arid and Semi-Arid Northwest China

**DOI:** 10.3390/plants13233412

**Published:** 2024-12-05

**Authors:** Xiran Sui, Qiongling Xu, Hui Tao, Bin Zhu, Guangshuai Li, Zengxin Zhang

**Affiliations:** 1 Joint Innovation Center for Modern Forestry Studies, College of Forestry and Grassland, College of Soil and Water Conservation, Nanjing Forestry University, Nanjing 210037, China; suixr@njfu.edu.cn (X.S.); koalaxu@njfu.edu.cn (Q.X.); binzhu@njfu.edu.cn (B.Z.); ligs@njfu.edu.cn (G.L.); 2 Xinjiang Institute of Ecology and Geography, Chinese Academy of Sciences, Urumqi 830011, China; taohui@ms.xjb.ac.cn

**Keywords:** vegetation dynamics vegetation recovery potential, vegetation cover, NDVI, warming and wetting climate, northwest China

## Abstract

The arid and semi-arid regions of northwest China are characterized by sparse vegetation and fragile ecosystems, making them highly susceptible to the impacts of climate change and human activities. Based on observed meteorological data, the Normalized Difference Vegetation Index (NDVI), the Lund–Potsdam–Jena dynamic global vegetation model (LPJ), a vegetation recovery potential model, and the MK trend test method, this study investigated the spatiotemporal distribution of vegetation recovery potential in northwest China and its relationship with global warming and increasing precipitation. The results indicated that vegetation in northwest China significantly increased, with greening closely related to trends in warming and wetting during 1982–2019. However, the vegetation recovery potential declined due to climate change. Central and southern Xinjiang and central Qinghai exhibited higher grassland recovery potential, while the central Gobi Desert areas of northwest China had lower recovery potential. The eastern part of northwest China was highly sensitive to drought, with moderate vegetation growth and recovery potential. Remote sensing data indicated a 2.3% increase in vegetation coverage in the region, with an average vegetation recovery potential index (IVCP) of 0.31. According to the results of LPJ model, the average vegetation recovery potential index for northwest China was 0.14, indicating a 1.1% improvement potential in vegetation coverage. Overall, climate warming and wetting facilitated vegetation recovery in northwest China, particularly in mountainous areas. The findings provide valuable insights for ecological restoration efforts and offer practical guidance for combating desertification and enhancing sustainable development. Moreover, these results underline the importance of incorporating vegetation recovery potential into regional policy-making to improve environmental resilience in the face of ongoing climate change.

## 1. Introduction

Vegetation restoration continues to stand out as one of the most potent strategies for mitigating climate change [1,2]. Moreover, vegetation restoration lies at the heart of ecological revitalization and combatting desertification [3]. Nevertheless, it was crucial to recognize that climate change will influence the prospects of natural vegetation recovery. By 2050, it was projected that the global potential canopy cover could decrease by approximately 223 million hectares, with the majority of these losses concentrated in tropical regions [2].

The majority of investigations into vegetation restoration potential predominantly consist of theoretical inquiries and empirical assessments of indicators. Traditional studies pertaining to vegetation restoration potential have primarily concentrated on qualitative analyses, relying heavily on expert knowledge and proving relatively inefficient. This limitation becomes especially evident when evaluating large-scale vegetation restoration efforts, necessitating the formulation of diverse indicators and corresponding weights for distinct regions [4,5].The precision of research and evaluation models for vegetation restoration was hampered by issues related to spatial heterogeneity and the absence of critical data points [3]. Traditional global-based evaluation models encounter challenges when applied to entire study areas, often leading to limited quantitative measurements [6,7,8], and only through quantitative assessments can the realistic value of restoration potentials be discerned.

Arid and semi-arid regions are highly sensitive to global climate change and are characterized by fragile ecosystems. Vegetation restoration in these areas is crucial not only for mitigating regional ecological degradation but also for contributing to global climate change mitigation, desertification control, and ecosystem recovery [9]. Vegetation restoration can offset some of the negative impacts of climate warming by increasing carbon sequestration, improving soil stability, and enhancing biodiversity [10]. As a typical arid and semi-arid region, the vegetation dynamics in northwest China directly influence the local ecological environment and socioeconomic development while also providing valuable references for similar environments globally [11]. In recent years, research on vegetation restoration in arid and semi-arid regions has gained momentum worldwide. For instance, studies on Central Asia, North Africa, and Australia have shown that vegetation restoration can effectively control desertification and enhance land productivity [9,12]. However, compared to these regions, northwest China exhibits unique climatic and geographical conditions, such as the combined effects of climate warming and humidification trends, complex terrain, and strong human intervention [13]. These factors make the processes and mechanisms of vegetation restoration in this region particularly distinctive [13,14]. Therefore, in-depth research on vegetation restoration potential in northwest China is not only vital for the sustainable development of its regional ecosystems but also provides valuable insights for the restoration and management of other ecologically vulnerable areas around the globe. Concerning the quantitative measurement of vegetation restoration potential [15], its essence lies in depicting the optimal conditions for vegetation growth attainable within the prevailing natural constraints of a given region. Xia [16] examined the maximum moisture-bearing capacity of vegetation in the soil. Han [17] used Loess Plateau of China as a case study to propose an ecological function-oriented vegetation protection and restoration framework based on PNV patterns and ecological functions. T. Li [18] formulates a composite index (E-j) based on comparing the trends of vegetation cover and vegetation productivity to assess ecological restoration effectiveness. Gao [19] calculated the restoration potential by borrowing the ecological moisture carrying capacity method, defined this potential as the restoration potential of vegetation cover in similar habitats, and evaluated the effects of forestry engineering measures through the division of similar habitats. Zhang [20] utilized the spatial sliding window technique to refine the model. Lv [4] introduced a model for assessing vegetation restoration potential at large spatial scales. They computed a vegetation restoration potential index and analyzed the optimal growth conditions achievable by vegetation once anthropogenic interference was removed. This analysis can serve as a scientific foundation for identifying key regions for restoring desertified grasslands in Mongolia and planning restoration initiatives. Zhou [21] concluded that the ecological environment in northwest China was susceptible and vulnerable to climate change, emphasizing that vegetation restoration can ameliorate the local ecological milieu. Duan [22] show that the annual occurrence frequency and annual days showed an upward trend before the 1980s and a significant downward trend after that, as well as significant turnarounds in the annual number of dust processes that occurred in the 1990s and around 2010, which revealed that vegetation cover effectively mitigates land desertification and the occurrence and impact of large-scale sandstorms. It is noteworthy that current studies often focus on individual factors, like vegetation or climate change [23,24], failing to provide a comprehensive assessment of climate change.

Despite research efforts spanning field investigations, climate ecological model simulations, remote sensing monitoring, and various other approaches, significant uncertainties persist in the findings of vegetation restoration potential research [20,21,25]. Ma et al. [26] analyzed the process of vegetation restoration in the eastern margin ecotone of the Qinghai–Tibet Plateau (EMEQTP) from 2001 to 2019 and showed that the NPP under a natural restoration mode had the highest increase, at 20.1%, and its spatial change showed an overall trend of restoration. Gu [27] showed that the average vegetation restoration potential in the study area was 0.837. Gao [14] established a vegetation response curve to analyze the future recovery trend of vegetation. Yi [5] employed a similar habitat method to project vegetation recovery potential in the Beiluo River basin. Zhang [28] reported that the forest, grassland, and desert ecosystems within the Three North Project region still possess a restoration capacity of 8.16%.

The mechanism used to calculate vegetation recovery potential in this study is based on the “similar habitat method”. In ecology, areas with potential for vegetation growth are often categorized based on similar habitats, meaning that locations with comparable natural conditions exhibit analogous landscapes. For instance, in regions with identical terrain, climate, soil, and hydrological factors, one would expect vegetation coverage to be similar as well. Any variations in these areas highlight the potential for additional growth. We refer to this potential as the vegetation recovery potential associated with similar habitats. Variations in vegetation recovery potential lead to differing levels of difficulty in vegetation restoration efforts; a higher recovery potential indicates an easier path to restoring vegetation, while a lower potential suggests more challenges [8].

The arid and semi-arid regions in northwestern China were shaped by their unique geography and ecosystems These areas face ecological sensitivity and meteorological risks, particularly severe droughts due to complex geographic and climatic factors [29]. In recent years, there have been numerous studies on the vegetation restoration potential in northwest China. For example, Wang [24] indicated that most vegetation-covered areas in the Hexi region experienced significant changes during the period of 2001–2017, revealing clear signs of regional vegetation deterioration. Hu [30] detected a significant improvement in vegetation recovery due to the reversion of farmland to forests and grasslands in northwest China. Also, it was found that runoff rates from the forested land were positively related to erosive rainfall (i.e., rainfall when runoff generated) and varied with forest canopy coverage. Zhang [31] identified an overall upward trend in vegetation cover in northwest China from 1982 to 2008. According to Shi [32], northwest China was shifting from a warm–dry to a warm–wet climate regime. The detrimental effects of this temperature increase have made climate change’s impact on northwest China a topic of extensive research [33]. However, studies addressing the potential for vegetation restoration under climate warming and humidification are still relatively scarce, especially quantitative ones.

In the past three decades, northwestern China has experienced consistent climatic warming and increased moisture levels. However, research on vegetation restoration potential in this region has been limited. Therefore, it is crucial to explore the potential for vegetative resurgence, particularly in arid and semi-arid regions, providing valuable insights for vegetation restoration efforts. This study seeks to shed light on the impact of these climatic changes on the recovery of native vegetation. Does the warmer and wetter climate facilitate or hinder vegetation restoration in northwestern China? Moreover, how do changes in vegetation distribution within the region respond to these alterations? This study is offers critical insights for ecological restoration, desertification control, and sustainable development. The findings underscore the importance of targeted ecological governance to address the challenges posed by environmental degradation in the arid and semi-arid areas of northwest China.

## 2. Results

### 2.1. Climate-Driven Vegetation Dynamics in Northwest China

**Figure 1:**
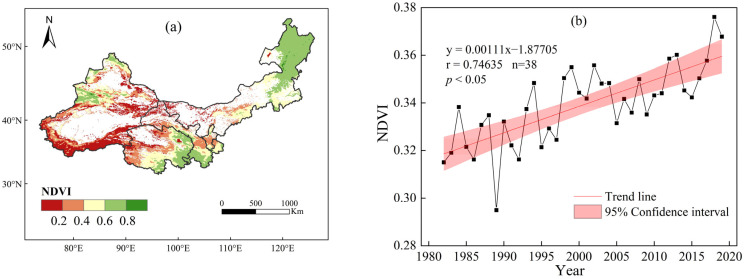

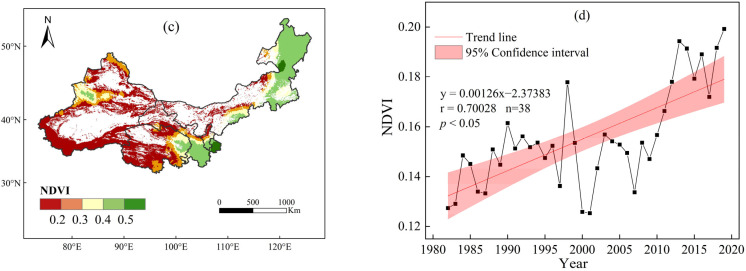
Trend of vegetation greening in northwest China during 1982–2019: (**a**), spatial distribution map of multi-year average satellite-observed NDVI; (**b**), annual variation in NDVI; (**c**), spatial distribution of multi-year average LPJ-simulated NDVI; (**d**), annual variation in NDVI.

This section utilizes NDVI data from AVHRR remote sensing imagery and NDVI data from LPJ simulations for vegetation greening analysis in the northwest. From 1982 to 2019, the regional average NDVI in northwest China showed a significant increasing trend (Figure 1b). The highest NDVI value was recorded in 2018 (0.38), and the lowest in 1989 (0.29). Spatially, vegetation coverage in northwest China was generally low (Figure 1a), with most areas characterized by sparse vegetation (NDVI < 0.4). These areas, accounting for 63.6% of the study region, were dominated by unused land types such as deserts and partially degraded grasslands. Regions with NDVI values between 0.4 and 0.6 were mainly located in central Qinghai, Gansu, Inner Mongolia, and Ningxia, as well as northwestern and central Xinjiang. These areas, covering 20.2% of the study region, were primarily cropland and some grasslands. Areas with high vegetation coverage (NDVI > 0.6) were found in northeastern Inner Mongolia, southeastern Qinghai, southern Gansu, and northwestern Xinjiang, where forests and grasslands were predominant, accounting for 16.2% of the region. According to LPJ model simulations, the regional average NDVI in northwest China also showed a significant increasing trend from 1982 to 2019, with the highest value in 2019 and the lowest in 2001 (Figure 1d). Regarding the spatial distribution of the grassland NDVI, the LPJ-simulated NDVI displayed spatial heterogeneity (Figure 1c), with higher NDVI values in the northeast, northwest, and southeast and lower values in the central region. Low NDVI values were concentrated around deserts, while high values were observed in the Tianshan Mountains, Daxinganling Mountains in Inner Mongolia, and Qinling Mountains.

**Figure 2:**
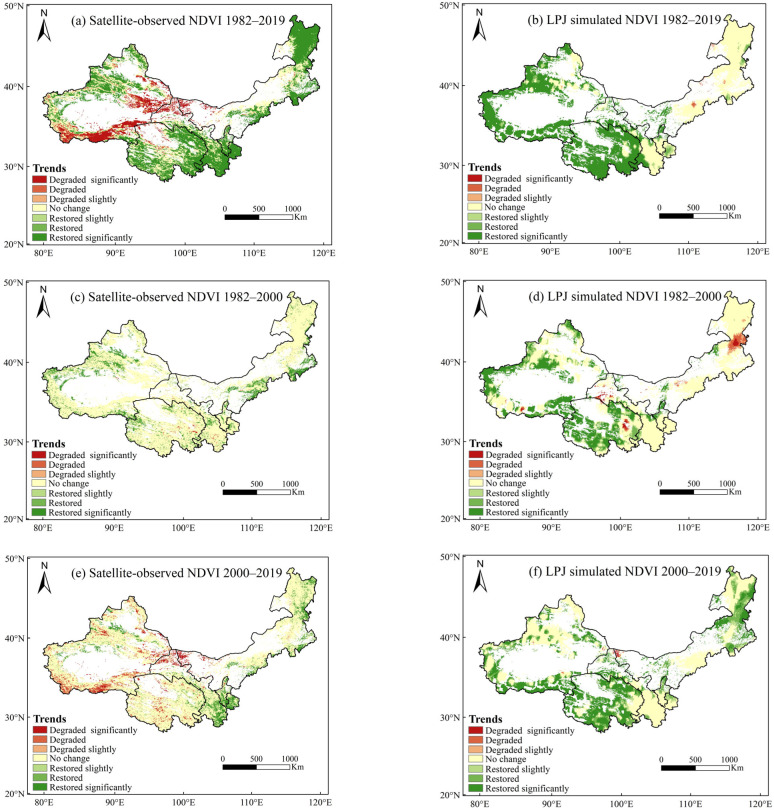
Changing trends of satellite-observed NDVI and LPJ-simulated NDVI in northwest China from 1982 to 2019. ((**a**), satellite-observed NDVI 1982–2019; (**b**), LPJ simulated NDV 1982–2019; (**c**), satellite-observed NDVI 1982–2000; (**d**), LPJ simulated NDVI 1982–2000; (**e**), satellite-observed NDVI 2000–2019; (**f**), LPJ simulated NDVI 2000-2019).

From 1982 to 2019, 33.82% of the total area in northwest China experienced a significant increase in the NDVI, 6.08% showed a moderate increase, and 2.8% displayed a slight increase (Figure 2a, Table 1). Figure 2c shows that during 1982–2000, NDVI trends were not significant, with only 20.63% of the regions exhibiting an increasing trend, and 78.36% showing no significant change. However, between 2001 and 2019, areas with a decreasing NDVI expanded to 24.13%, while areas with an increasing NDVI accounted for 16.18%, indicating simultaneous vegetation restoration and degradation after 2000 (Figure 2e). Areas with an increasing NDVI from 1982 to 2019 were mainly located in eastern and southern Inner Mongolia, Ningxia, southern Gansu, eastern and western Qinghai, and northern and western Xinjiang. In contrast, southern Xinjiang, northern Qinghai, northern Gansu, and western Inner Mongolia showed a declining trend, with 21.3% of the regions experiencing a significant decrease in the NDVI. The LPJ simulation results showed that from 1982 to 2019, 52.39% of the total area in northwest China had a significant increase in the NDVI, 5.63% showed a moderate increase, and 3.04% displayed a slight increase (Figure 2b, Table 2). Figure 2d indicates that during 1982–2000, NDVI trends were not significant, with only 40.81% of the regions exhibiting an increasing trend, while 54.4% showed no significant change. However, between 2001 and 2019, areas with a decreasing NDVI accounted for 0.62%, while areas with an increasing NDVI accounted for 62.72%, indicating substantial vegetation recovery after 2000 (Figure 2f). Areas with an increasing NDVI from 1982 to 2019 were primarily located in eastern and western Qinghai and northern and western Xinjiang. In contrast, eastern Inner Mongolia showed a declining trend, with 0.2% of the regions experiencing a significant decrease in the NDVI.

### 2.2. Evolution of Vegetation Restoration Potential in Northwest China

**Figure 3:**
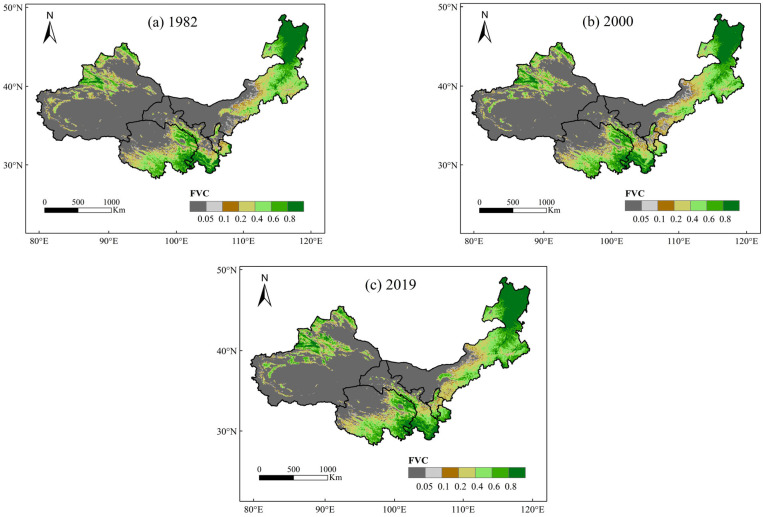
Spatial distribution map of satellite-observed vegetation coverage in 1982 (**a**), 2000 (**b**), and 2019 (**c**).

Based on NDVI data from AVHRR remote sensing imagery, a two-dimensional pixel model was computed to obtain vegetation cover (FVC) data from 1982 to 2019; the years 1982, 2000, and 2019 were specifically chosen for vegetation cover analysis, resulting in the generation of spatial distribution maps representing vegetation cover. Figure 3 illustrates that, in general, vegetation cover in northwest China has experienced a positive trend over the years. This trend was predominantly characterized by a gradual transition from areas with low vegetation cover to those with high vegetation cover in certain regions. Additionally, there was an expansion of high vegetation cover areas from the central region towards the periphery. This notable increase in vegetation coverage was primarily concentrated in the northeastern part of Inner Mongolia, northwestern Xinjiang, eastern and southern Qinghai, and southern Gansu. However, it is important to note that significant portions of the central northwest still exhibit low vegetation coverage.

By reclassifying the fractional vegetation cover (FVC) for northwest China in 1982, 2000, and 2019 and calculating the proportion of area covered by each vegetation category, the time-varying characteristics of vegetation cover were analyzed (Table 3). Combined with the characteristics of the study area, the vegetation cover was classified into seven grades, namely, very low cover (FVC < 0.05), low cover (0.05 < FVC < 0.1), lower cover (0.1 < FVC < 0.2), medium–low cover (0.2 < FVC < 0.4), medium cover (0.4 < FVC < 0.6), medium–high cover (0.6 < FVC< 0.8), and high coverage (0.8 < FVC < 1). As can be seen from the table, the vegetation cover in northwest China from 1982 to 2019 increased in high cover and medium–high cover areas, of which the largest increase was observed for medium–high cover, which increased by 2.68%. Medium–low cover first increased significantly and then slightly decreased, increasing overall. Vegetation cover for medium–low cover remained basically unchanged and decreased for very low, low, and lower cover; specifically, from 1982 to 2019, it decreased by 3.65%, 1.12%, and 0.49%, respectively. Overall, there was a significant improvement in vegetation cover in northwest China from 1982 to 2019.

In this part, FVC calculated from NDVI data from AVHRR remote sensing images and FVC calculated from NDVI data from LPJ simulations are used to study and analyze the vegetation restoration potential of the northwest region by using the vegetation restoration potential calculation model.

**Figure 4:**
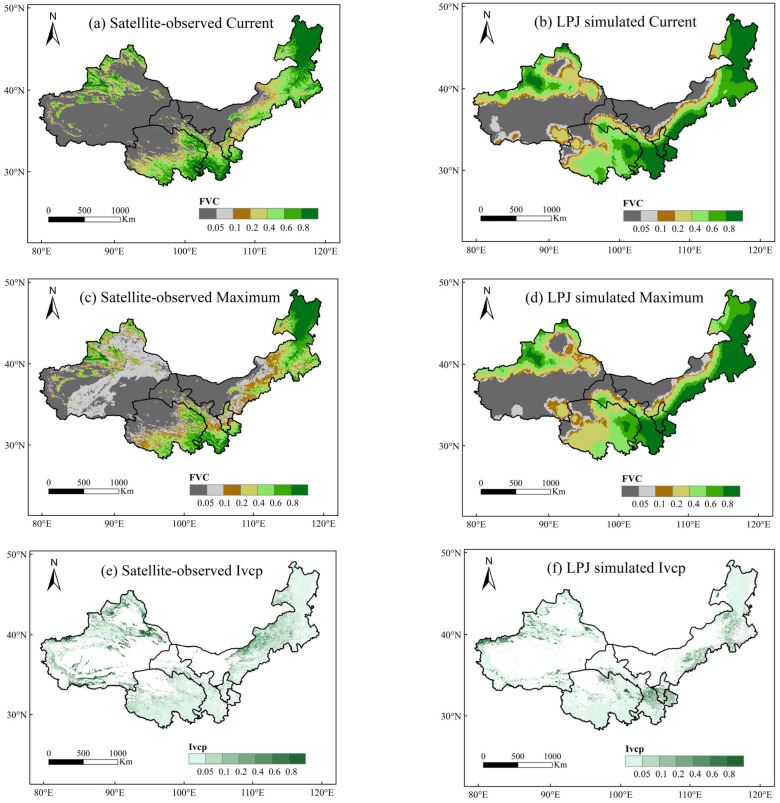
Current vegetation cover, maximum recovery cover, and natural vegetation restoration potential index in northwest China from 1982 to 2019 based on satellite-observed data and LPJ simulated data. ((**a**), satellite-observed Current; (**b**), LPJ simulated Current; (**c**), satellite-observed maximum; (**d**), LPJ simulated maximum; (**e**), satellite-observed Ivcp; (**f**), LPJ simulated lvcp).

According to the remote sensing data, the spatial distribution of vegetation cover in 2019 in the northwest China region varied significantly (Figure 4a), with higher vegetation cover in eastern Inner Mongolia, southern Qinghai, western Xinjiang, and southern Ningxia, and an average of 21% vegetation cover in 2019 in northwest China. The maximum recovery cover of future average vegetation determined by the “similar habitat” method was 23.3% (Figure 4c), which meant that there was still an estimated 2.3% improvement in vegetation cover in the region.

To quantify the disparity between current vegetation growth and its potential for recovery, a model for calculating the natural recovery potential index of vegetation was employed (Equation (5)). The initial breakpoint value, representing the maximum vegetation cover threshold within the spatial window, was determined using the natural breakpoint method, thus yielding its spatial distribution. The vegetation recovery potential index was categorized into five levels ranging from low to high [34], where a higher index indicates higher potential. Figure 4e shows that the potential is low in the desert, Gobi, and saline areas; it is also low in the eastern and southern parts of northwest China, which results from the high percentage of primary vegetation cover in these areas, as well as being high in grassland–meadow areas in northwest Xinjiang and central Qinghai. These findings suggest that this area has the potential to mitigate the arid and semi-arid conditions prevailing in the region. In summary, the study area as a whole demonstrates little potential for vegetation restoration, and the mean vegetation restoration potential index of northwest China was 0.31.

Based on the NDVI data from 1961 to 2019 in northwest China simulated by the LPJ model, the spatial distribution of vegetation cover in 2019 was simulated (Figure 4b), with an average cover of 34.2% in 2019, and the spatial distribution of the maximum vegetation cover was simulated (Figure 4d), with a maximum projected average cover of 35.3% in the future. This led us to estimate that there was still a 1.1% improvement in vegetation cover in the region. As a large-scale model, the simulation results of the LPJ model have obvious spatial heterogeneity, which was, in general, in line with that of remote sensing. From the map, it can be seen that the vegetation cover was higher in the northeast, northwest, and southeast of northwest China and low in the center, mainly concentrated at 0.2.

The spatial distribution of simulated vegetation that could be restored by LPJ simulation was obtained by using the vegetation restoration potential index (Equation (5)), and the results are shown in Figure 4f, which is generally consistent with the remote sensing results as a whole, with an average vegetation restoration potential index of 0.14 (Table 4).

**Figure 5:**
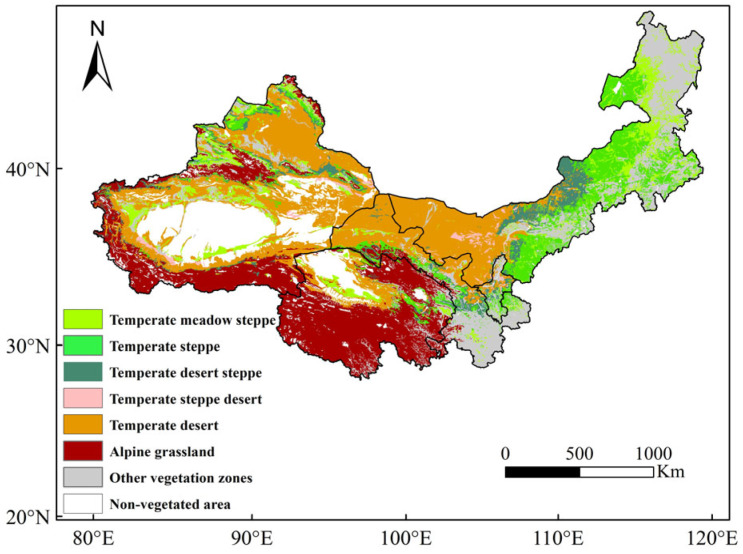
Atlas of grassland resources in northwest China.

Vegetation types in northwest China are dominated by grasslands (Figure 5), with temperate deserts in the central part of the study area, forests and farmlands outside in the east, alpine grasslands at high altitudes such as in the south and central part of the study area, and temperate meadow grasslands, temperate typical grasslands, and temperate desertified grasslands in the other areas.

**Figure 6:**
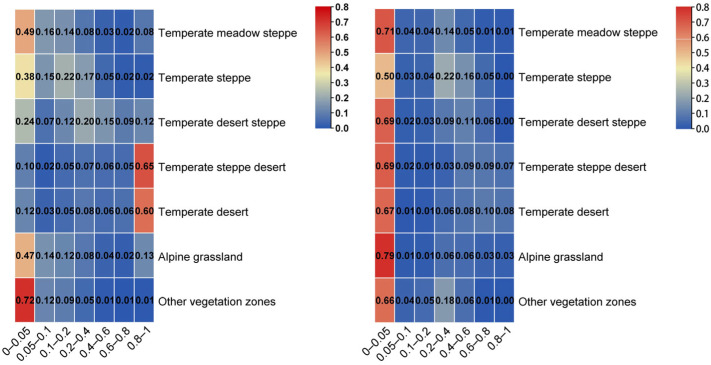
Vegetation restoration potential indices under different grassland types simulated by satellite observation (**left**) and the LPJ model (**right**).

Using zonal statistics, the vegetation recovery potential index was analyzed under different grassland types. The results of the remote sensing data indicated that the vegetation recovery potential index of temperate steppe and desert was higher, which was manifested by the fact that more than 70% of these areas had an IVCP > 0.4, and the area with an IVCP < 0.05 had a lower index (Figure 6). The results of the LPJ model indicated that the vegetation recovery potential index of temperate steppe was higher, which was evident through the proportion of the area with an IVCP > 0.4 being greater than 20% and the proportion of the area with an IVCP < 0.05 being lower (Figure 6).

**Figure 7:**
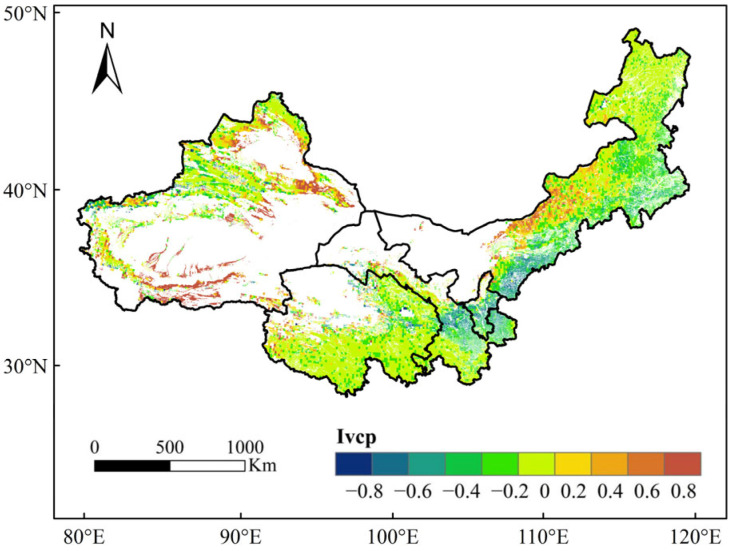
Differences in vegetation restoration potential index between LPJ-simulated maximum recovery cover and satellite-observed current vegetation cover in northwest China from 1982 to 2019.

We analyzed the differences between the remote sensing data and the vegetation natural recovery potential simulated by the LPJ model using the residual method based on ArcGIS 10.8 software, and the results are shown in Figure 7. The difference between the two was small, mostly between −0.2 and 0.2; the areas greater than 0.6 were mainly the southern and northern parts of the edge of the Taklamakan Desert; and the areas less than −0.6 were mainly the central–southern part of Gansu and the central part of Ningxia.

### 2.3. Response of Vegetation Restoration Potential to Climate Warming and Humidification

**Figure 8:**
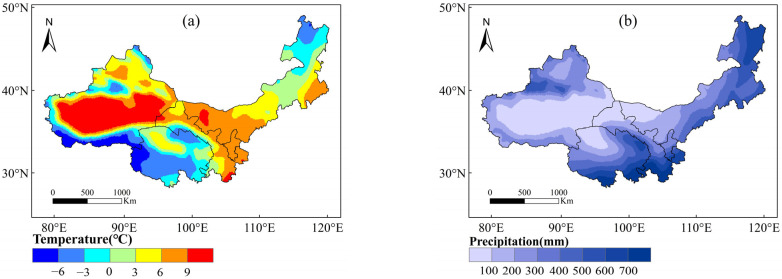
Spatial distribution of station-measured annual mean temperature (**a**) and precipitation (**b**) in northwest China during 1961 to 2019.

Based on historical temperature and precipitation data collected from meteorological stations between 1961 and 2020, the spatial analysis results for northwest China are as follows: the temperature distribution exhibited a distinct westward-high, eastward-low pattern, as well as a northward-high, southward-low trend. Areas characterized by the highest temperatures were primarily concentrated in expansive regions encompassing central Xinjiang, southeastern Gansu, Ningxia, and western Inner Mongolia. In contrast, lower temperatures prevailed in the southern parts of Xinjiang, extensive areas of Qinghai, and northeastern Inner Mongolia. Notably, the temperature variability across the study area exhibited considerable spatial heterogeneity (Figure 8).

Regarding precipitation, the overall distribution showcased elevated levels in the northwestern and southeastern fringes of the region, juxtaposed with a marked precipitation deficit in the central expanse. Regions with heightened precipitation were chiefly clustered in the northwestern sector of Xinjiang, Qinghai, the southern part of Gansu, and the eastern and northern sectors of Inner Mongolia. Conversely, areas with diminished precipitation were predominantly situated in the central portion of Xinjiang, as well as border regions shared with other provinces (Figure 8).

**Figure 9:**
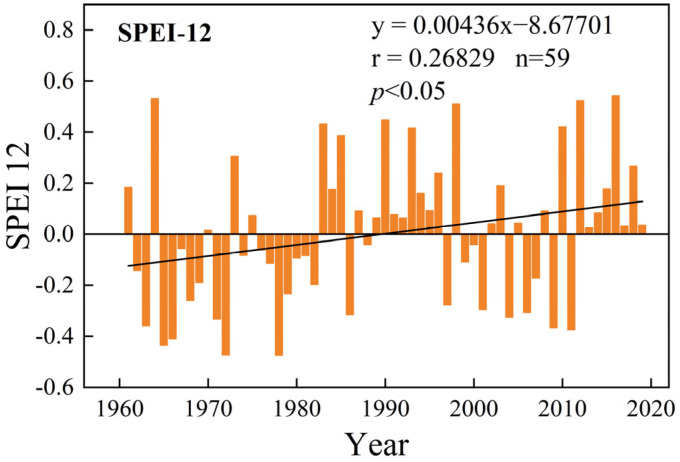
Changes in Standardized Precipitation Evapotranspiration Index (SPEI) at 12-month scale in northwest China from 1961 to 2019.

Studies have shown that precipitation is the primary factor affecting vegetation in northwest China, and based on this, it is necessary to analyze the aridity index (SPEI) in northwest China. The SPEI, a drought index, exhibits notable variations in volatility across different time scales. These variations in the time-scale length and frequency of fluctuations display a consistent pattern, highlighting the strong stability of longer time scales. In this study, the SPEI-12 index was chosen to illustrate the evolving climate trends of warming and humidification in northwest China from 1961 to 2019 (Figure 9). It can be observed that northwest China experienced a higher frequency of drought events during the periods of 1961–1986 and 1997–2011. Conversely, during the periods of 1986–1997 and 2011–2019, the occurrence of humid events became more prevalent, indicating an overall shift from dry to wet conditions. Therefore, in conjunction with the increasing temperature trend, it becomes evident that the climate in northwest China has undergone a significant pattern of warming and humidification.

**Figure 10:**
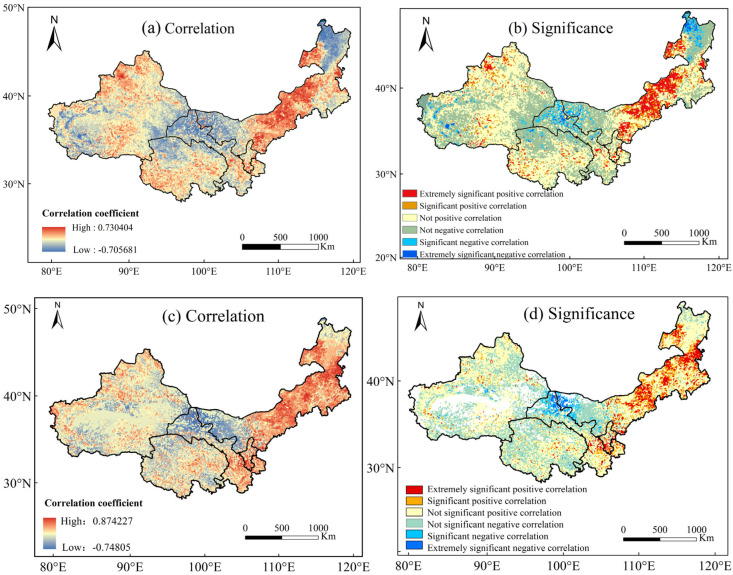
(**a**,**b**) represent the correlation and significance between NDVI and SPEI-12 in northwest China from 1982 to 2019; (**c**,**d**) represent the correlation and significance between FVC and SPEI-12 in northwest China from 1982 to 2019.

Precipitation is the most important natural factor limiting vegetation growth in the study area [35]. To investigate the response of vegetation conditions to drought conditions, the spatial correlation and significance of the multi-year mean values of the NDVI and SPEI-12 from 1982 to 2019 were analyzed. As shown in Figure 10a, the response of annual vegetation growth to the annual-scale drought index SPEI-12 was high, with a maximum correlation coefficient of 0.73, a minimum correlation coefficient of −0.71, and a mean correlation coefficient of 0.06. Higher correlation coefficients were mainly distributed in central Inner Mongolia, northwestern Xinjiang, Qinghai, and northern Gansu, and lower correlation coefficients were mainly distributed in western Inner Mongolia, southwestern Xinjiang, northern Qinghai, and northern Gansu. As shown in Figure 10b and Table 5, the annual NDVI and the annual-scale SPEI-12 in most of the regions showed a non-significant correlation, accounting for 81.08%, with the positive correlation being more non-significant than the negative correlation. The percentage of areas showing significant and highly significant positive correlations (13.89%) is also higher than that of areas with a negative correlation (5.03%) at *p* < 0.05. There was an overall positive correlation between NDVI and SPEI-12 in the study area from 1982 to 2019 (59.2%).

The correlation analysis between the annual NDVI and SPEI-12 showed that the correlation between grassland and drought was the most significant, the positive correlation between farmland and drought was only lower than that of grassland, and the correlation between forest and drought was not significant or even negative, which indicated that forests were weakly affected by drought, that the sensitivity of vegetation to drought response varied among the different land cover types, and that the growth status of grassland was more indicative of drought status (Figure 10a,b).

The larger the SPEI value, the more humid the conditions; conversely, the smaller the value, the drier the conditions. On an annual scale, the correlation between FVC and SPEI-12 revealed that in the eastern part of northwest China, namely eastern Inner Mongolia, central and southern Ningxia, and central Gansu, FVC was positively correlated with SPEI-12. This means that higher SPEI values, indicating lower drought levels, corresponded to higher FVC. In contrast, FVC and SPEI-12 showed a significant negative correlation in northern Gansu and the northwest corner of Inner Mongolia. This may be due to the widespread distribution of underlying surfaces, where greater evapotranspiration leads to the loss of more soil moisture, thereby inhibiting vegetation growth (Figure 10c,d).

In order to investigate the dynamic response of vegetation restoration potential to climate warming and humidification, spatial and temporal variations in maximum vegetation cover (FVC), or peak vegetation cover, were analyzed from 1982 to 2019. The process for obtaining the peak value of vegetation cover is to use the maximum synthesis method to obtain the maximum vegetation cover through the vegetation cover data from each year from 1982 to 2019 and then to use the maximum vegetation cover to subtract the raster of the vegetation cover of each year. Then, the number of rasters whose value is equal to 0 indicates the peak value of vegetation cover for each year, so as to obtain the temporal change in the peak value of vegetation cover. The latitude and longitude of a raster with a value equal to 0 were extracted to obtain the spatial variation in the peak value of vegetation cover.

**Figure 11:**
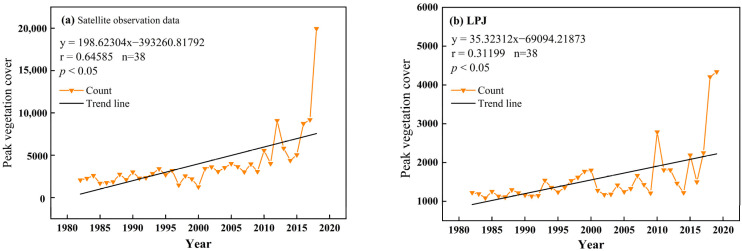
Temporal changes in peak vegetation cover in northwest China, 1982–2019 ((**a**), satellite observation data; (**b**), LPJ model simulation).

Based on the satellite observation data, the temporal changes in the maximum vegetation cover (FVC), i.e., the peak vegetation cover, in northwest China from 1982 to 2019 were obtained. The results, as shown in Figure 11a, show that the peak vegetation cover in northwest China from 1982 to 2019 shows a fluctuating upward trend, with the maximum value occurring in 2019 with a value of 19,956 and the minimum value occurring in 2000 with a value of 1259. Based on the data simulated by the LPJ model, the temporal changes in the peak vegetation cover in northwest China from 1982 to 2019 were obtained. The results are shown in Figure 11b, the peak vegetation cover in northwest China from 1982 to 2019 showed a fluctuating upward trend, with the maximum value appearing in 2018 and the minimum value appearing in 1984.

It can also be seen from the figures that the maximum values of vegetation cover after 2000 are, overall, higher than the maximum values of vegetation cover before 2000. This indicates that vegetation recovery may be affected by climate warming and humidification, with a fluctuating upward trend in the vegetation cover maximum and a downward trend in the vegetation recovery potential index.

**Figure 12:**
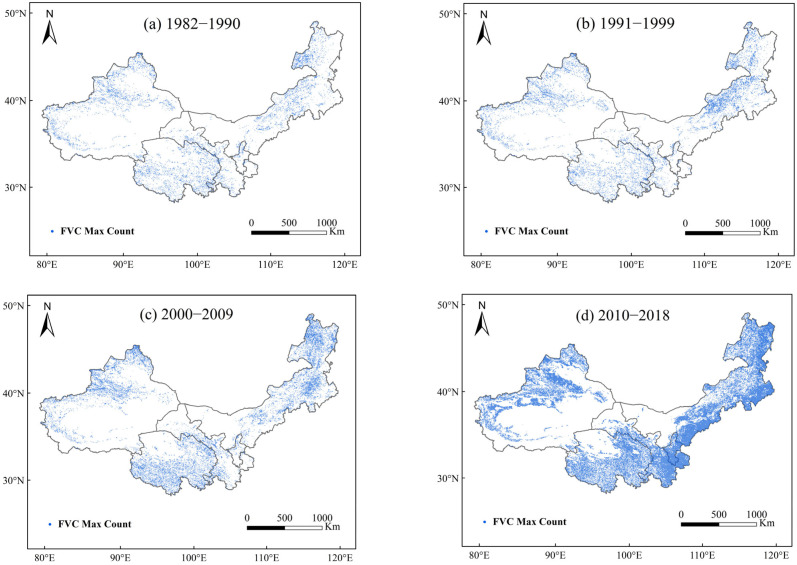
Spatial variation in maximum fractional vegetation cover (FVC) in northwest China from 1982 to 2019 using satellite-observed data ((**a**), 1982–1990; (**b**), 1991–1999; (**c**), 2000–2009; (**d**), 2010–2019).

**Figure 13:**
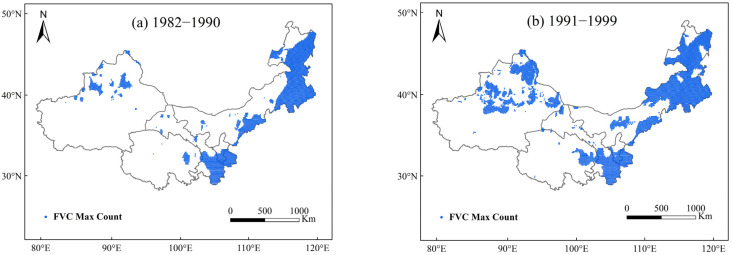

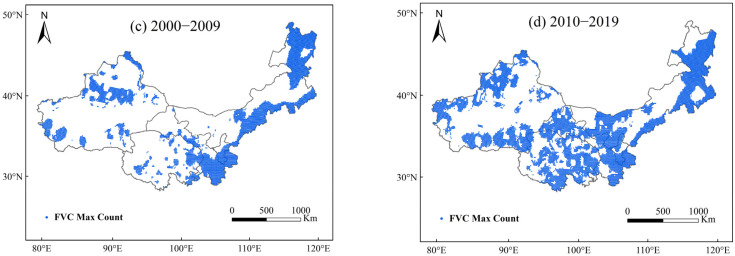
Spatial variation in maximum fractional vegetation cover (FVC) in northwest China from 1982 to 2019 using LPJ model simulation ((**a**), 1982–1990; (**b**), 1991–1999; (**c**), 2000–2009; (**d**), 2010–2019).

Using satellite observation data and LPJ model simulation data, the spatial changes in the maximum vegetation fractional cover (FVC) in northwest China in different years from 1982 to 2018 were obtained. The results shown in Figure 12 and Figure 13 indicate that the maximum vegetation cover values in northwest China from 1982 to 2018 exhibited a clear upward spatial trend, with the most significant changes occurring from 2010 to 2018 and the least significant changes occurring from 1982 to 1990. This indicates that the vegetation growth shows a favorable trend.

## 3. Discussion

### 3.1. Characteristics of Vegetation Greening in Northwest China

This study found that the NPP and NDVI of vegetation in northwest China generally showed an increasing trend during 1982–2019, consistent with the findings of Zhang et al [11]. A significant shift in the NDVI was observed in 1999, with post-1999 increases being more significant than those before 1999. Vegetation changes in northwest China exhibited clear spatial heterogeneity. Both the NDVI and LPJ-simulated NDVI indicated that the areas showing increases were primarily located in ecologically sensitive regions, including eastern and southern Inner Mongolia, Ningxia, southern Gansu, southeastern Qinghai, and northwestern Xinjiang. In contrast, a decreasing trend was observed in southern Xinjiang, northern Qinghai and Gansu, and western Inner Mongolia. The overall trend in vegetation cover in northwest China showed positive growth, with significant increases mainly concentrated in northeastern Inner Mongolia, northwestern Xinjiang, eastern and southern Qinghai, and southern Gansu. However, it is worth noting that the central desert regions of northwest China still had very low vegetation coverage.

In addition to human activities, changes in vegetation cover in northwest China are mainly influenced by topography and the regional climate [36,37]. In areas close to mountains, the high base level of precipitation and the warming and humidifying climate favor vegetation improvement [38]. In desert regions, however, temperature increases outweigh precipitation increases [39]. The rise in temperature leads to higher evaporation rates, which offset the benefits of increased precipitation, resulting in insignificant vegetation growth or even degradation [40]. Furthermore, in the context of global warming, melting glaciers are affecting vegetation in areas through which meltwater flows [41]. Increased precipitation or meltwater can enhance grass growth, making it taller and more lush [42], a phenomenon particularly evident in the Ili region of Xinjiang. In the Ili region, western Northern Xinjiang, and eastern Gansu, agricultural areas have the potential to expand, and water resource issues have been alleviated. However, these regions’ capacity to handle increased precipitation is limited. Excessive rainfall can have adverse effects, significantly impacting agricultural production and ecosystem growth. This is especially true for certain crops and livestock farming, which may be notably affected by changes in precipitation patterns [43].

### 3.2. Dynamic Response of Vegetation Recovery Potential to Climate Change in Northwest China

In the context of global climate warming and the warming and humidification of northwest China, this study found that the overall potential for vegetation restoration in the region is declining. Spatially, it was observed that the western part of northwest China primarily consists of rotational grazing zones [44]. This area is characterized by high ecosystem fragility, severe degradation, and a high potential for vegetation restoration with ample restoration space. This region is adjacent to the southwestern Gobi Desert, making it susceptible to the encroaching effects of desertification [45]. In the central region, which mainly consists of sand control and desertification prevention areas, desertification is extremely severe, and the potential for vegetation restoration is low, as the climate conditions are not conducive to vegetation growth. In the eastern region, which is primarily characterized by scientific grazing and artificial grassland construction, vegetation growth is highly sensitive to drought. The current state and potential for vegetation growth are moderate. This area could moderately develop agriculture and animal husbandry practices, but the ecosystem is extremely sensitive. Climate fluctuations or improper land use could severely damage the ecosystem, potentially leading to a shift in land cover from grassland to desertified grassland or even desert [11].

The spatial differences in vegetation restoration potential are primarily attributed to the shift in northwest China from a “warm–dry” to a “warm–wet” climate [46]. This transition began in the 1980s, when the warming Atlantic currents started to strengthen, leading to increased warm and moist airflows. This change is part of the interdecadal variability of oceans [14]. Since 2010, this trend has intensified along with Arctic warming, resulting in an increased transport of moisture and heat from the Arctic to lower latitudes [47]. In reality, influenced by atmospheric circulation, the warm–wet trend in northwest China is extending westward into Central Asia and eastward into western North China [48]. The warm–wet trend is most pronounced from China and Central Asia to northern India and Pakistan. Such extensive warming and moistening across regions from North Africa through West Asia to Central Asia and East Asia would not be possible without the concurrent intensification of the Atlantic warm currents and Arctic warming [49]. Currently, the warm–wet trend is having a positive effect on most of northwest China, leading to overall improvements in vegetation [50]. However, whether this greening trend will continue or increased potential evaporation in drier areas will result in greater aridity remains uncertain. Future scenarios will be explored using the LPJ model to simulate different outcomes.

In the western grassland rotational grazing areas of northwest China, it is essential to focus on wind and sand control measures [51]. This includes installing grass grids, sand barriers, and sand control nets and establishing windbreak and sand control forests using a combination of biological, engineering, and chemical methods to prevent the spread of eastern deserts to the west. In the central region, which is primarily involved in sand control and reclamation projects, emphasis should be placed on wind and sand prevention measures, primarily using engineering and chemical approaches. These measures will help prevent the spread of desertification and the intensification of dust storms, potentially impacting surrounding areas [52]. Some degree of vegetation restoration measures can be implemented, integrating biological and engineering methods. In the eastern region, attention should be given to climate fluctuations. When signs of drought begin to appear, it is advisable to implement grazing restrictions and rotational grazing policies. During severe drought conditions, artificial water supplementation and ecological protection measures should also be considered [53,54].

Finally, regarding the limitations and future prospects of this study, we recognize that while the LPJ model and satellite observations provide essential scientific support for vegetation dynamics research, they also have certain limitations. For example, the LPJ model relies on several assumptions to simulate vegetation–climate interactions, which may oversimplify complex ecological processes, particularly in regions with high spatial heterogeneity like northwest China. Similarly, satellite observation data are influenced by atmospheric conditions, sensor limitations, and resolution constraints, which may lead to inaccuracies in capturing fine-scale vegetation changes. These uncertainties highlight the need to integrate multi-source data and improve model calibration methods to enhance the reliability of the research findings.

## 4. Materials and Methods

### 4.1. Study Area

**Figure 14:**
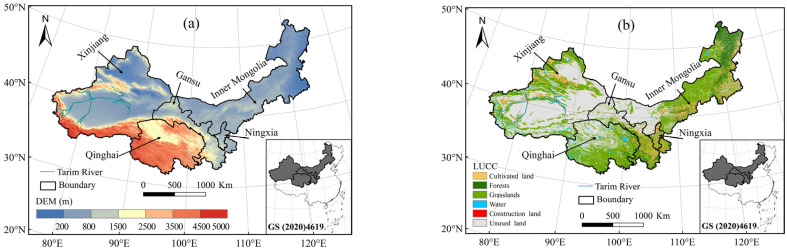
Study area (**a**) and distribution map of land use (**b**). Note: this study used the national standard map of China, with the Map Approval Number China_GS (2020)4619 without any modifications.

The arid and semi-arid regions of China located in the northwest, north of the Great Wall and Kunlun Mountains, including Xinjiang, Inner Mongolia, northern Ningxia, western Gansu, and the Qaidam Basin in Qinghai. These areas cover about one-third territory of China. To maintain administrative consistency, this study selects five provinces or regions namely Xinjiang,, Gansu, Inner Mongolia, Ningxia, and Qinghai—referred to as the northwestern region. The study area spans 31°36′ N to 53°23′ N and 73°40′ E to 126°04′ E, covering approximately 4.06 million km^2^ (Figure 14a) [55]. The northwestern region selected for this study spans three of topographic steps of China, featuring a wide range of elevations and complex landforms. The main terrains in this area include plateaus, basins, and mountains, such as the Qinghai–Tibet Plateau, Loess Plateau, Inner Mongolia Plateau, Tarim Basin, Qaidam Basin, Junggar Basin, Tianshan Mountains, Kunlun Mountains, and Qilian Mountains [56]. The northwestern region is located in the hinterland of Eurasian continent,, making it difficult for moist air from the oceans to reach it. The region is influenced by the interaction between the summer monsoon, westerlies, and plateau winds, resulting in a predominantly continental monsoon climate. It is characterized by aridity, low rainfall, long sunshine duration, high evapotranspiration, frequent sandstorms, and significant dryness. As a result, the region faces severe water scarcity and fragile ecosystems, making it not only a typical resource-limited area but also one of the regions most sensitive to global climate change.

### 4.2. Data Collection and Processing

#### 4.2.1. Vegetation Data

The NDVI dataset employed in this investigation was sourced from the 5 km resolution monthly NDVI grid dataset of China, accessible via the National Science and Technology Resource Shared Service Platform—National Earth System Science Data Center (http://www.geodata.cn, accessed on 1 March 2023). The temporal scope encompasses the years 1982 to 2019. This dataset was meticulously generated through the processes of monthly synthesis, mosaicing, and cropping, leveraging the NASA AVHRR CDR NDVI V5 version, which offers day-to-day data. The Advanced Very High Resolution Radiometer (AVHRR), a pivotal instrumentation component of the NOAA satellite series, serves as the primary means of observation. Being noteworthy for its extended revisit cycle, the AVHRR allows for daily monitoring of actual vegetation dynamics, capturing the intricate interplay between human activities and natural influences on vegetation evolution. To mitigate the impact of atmospheric variables such as cloud cover, aerosols, cloud shadows, viewing angles, solar altitude, and extreme monthly values, this study employs the Maximum Value Composition (MVC) methodology. This approach selects the maximum NDVI value from each month to represent the annual NDVI data. Subsequently, the ENVI tool was employed to compute vegetation cover.

FVC is usually constructed using an index to construct a dimidiate pixel model, which is the simplest and most commonly used model for linear pixel decomposition assuming that the image pixel is composed of two components, non-vegetation and vegetation, and the spectral information is linearly combined by them. The FVC value of each pixel is obtained by calculating the proportion of vegetation for the pixel, and the formulae are as follows [57]:

(1) $$FVC=\left(NDVI-{NDVI}_{min}\right)/\left({NDVI}_{max}-{NDVI}_{min}\right)$$

where FVC is the vegetation cover; NDVI is the vegetation index of the requested image; ${NDVI}_{max}$ and ${NDVI}_{min}$ are the maximum and minimum values of NDVI in the study area, respectively; and the vegetation cover was calculated using the ENVI tool with a 95% confidence interval and a 5% vegetation index.

#### 4.2.2. Meteorological Data

This study employs observational data encompassing monthly gridded temperature and precipitation, with a spatial resolution of 0.5° × 0.5° (http://data.cma.cn/site/index.html, accessed on 31 January 2020), sourced from 2472 meteorological stations in China spanning the period 1961–2019. Within this investigation, meteorological parameters, including precipitation, maximum temperature, minimum temperature, relative humidity, wind speed, and sunshine duration, were procured from the daily dataset of fundamental meteorological elements (V3.0) derived from China’s national surface weather stations, as well as the monthly data originating from surface climate of China (http://data.cma.cn/, accessed on 12 September 2022). Meteorological stations exhibiting missing data, accounting for more than 5% of the time series, were subjected to exclusion, and the gaps in meteorological data were rectified through linear interpolation.

#### 4.2.3. SPEI Drought Index Data

The Standardized Precipitation Evapotranspiration Index (SPEI) is an internationally used drought index, and the data source is a globally gridded dataset (https://spei.csic.es, accessed on 20 November 2023), SPEI base v2.9, based on monthly precipitation and potential evapotranspiration from the CRUTS4.07 dataset. Spanning from January 1901 to December 2022, the dataset has a time scale of 1 to 48 months and a spatial resolution of 0.5°.

The calculation procedure is mainly based on the national standards for meteorological drought levels [58]. The SPEI is designed to take into account both precipitation and potential evapotranspiration (PET) in determining drought. Thresholds, i.e., ranges of values for different levels of classification criteria for the SPEI, a drought index, are first determined, then potential drought events are identified, and finally, drought events are filtered out from potential drought events. In this paper, continuous processes with SPEIi ≤ −0.5 and SPEIi + 1 > −0.5 are chosen as drought events.

Drought frequency describes the rate of drought occurrence at a specific location or grid point over a period. The higher the value, the higher the frequency of drought. The calculation formula is as follows:

(2) $$P=\frac{n}{N}\times 100\%$$

In the formula, P is the drought frequency, n is the total number of months or years with drought at the station or grid point, and N is the total length of the data series for the station or grid point (Table 6).

### 4.3. Model and Methodology

#### 4.3.1. LPJ Model

The LPJ (Lund–Potsdam–Jena) model, as a dynamic global vegetation model (DGVM), can simulate the vegetation distribution and carbon and water exchange processes in ecosystems [15,59]. Vegetation distribution in the LPJ model was based on bioclimatic indicators, and carbon was stored in leaves, roots, heartwood, sapwood, and above-ground and below-ground apoplastic pools, as well as in two soil carbon pools [60]. The LPJ model simulates the carbon and water cycles and acts out the terrestrial carbon and water exchange processes one by one on a grid basis. The water cycle module of the model takes into account precipitation, snowmelt, runoff, vegetation retention, and vegetation transpiration to simulate vegetation–soil–atmosphere water exchange. The carbon cycle in the model mainly takes into account the processes of respiration, photosynthesis, the decomposition of fallen material, and the growth and death of vegetation and introduces the perturbation mechanism of fire, thus simulating the flow of carbon dioxide between vegetation, soil, and the atmosphere [61,62].

The input data for the LPJ model were monthly average temperature, monthly precipitation, monthly cloudiness, monthly wet days, soil texture, CO_2_ concentration, etc. The output data were net primary productivity, leaf area index, soil carbon stock, biomass, evapotranspiration, runoff, etc. In the LPJ model, the NPP, leaf area index, soil carbon stock, biomass, evapotranspiration, and runoff were all used [63]. In the LPJ model, NPP (net primary productivity) was calculated by subtracting the amount of carbon consumed by growth respiration and maintenance respiration from the Gross Primary Production (GPP), which was calculated by coupling photosynthesis and water balance schemes [64]. The formula for calculating NPP was as follows:

(3) $$NPP=0.75\times \left(GPP-R_{m}\right)$$

where GPP was the TPP (Total Primary Productivity) in gC-m^−2^-y^−1^; $R_{m}$ was the amount of carbon required for maintenance respiration in gC-m^−2^-y^−1^; and 0.75 means that 25% of $GPP-R_{m}$ was consumed for vegetative growth and maintenance respiration.

#### 4.3.2. Vegetation Restoration Potential Methodology

In general, areas with similar natural conditions have similar landscape and vegetation cover [4,19]. When confronted with uniform topographic parameters such as elevation, slope, and aspect, along with climatic factors including precipitation, temperature, and humidity, it was foreseeable that the natural landscape and vegetation cover would display congruence. Any variance in vegetation cover detected within the same range of natural conditions signifies an inherent potential, enabling the computation of localized vegetation restoration potential [65]. In order to determine the optimal vegetation growth under natural conditions, the indeterminate window method was used to construct similar habitats [66].

(4) $${VRP}_{ij}={NDVI}_{kl}\left(V_{1},V_{2}\cdots V_{n}\right)max$$

where ${VRP}_{ij}$ denotes the vegetation restoration potential in row i and column j; ${NDVI}_{kl}\left(V_{1},V_{2}\cdots V_{n}\right)max$ represents the maximum NDVI in the window where the point was located; and $V_{1},V_{2}\cdots V_{n}$ represent n environmental variables.

#### 4.3.3. Model for Calculating the Vegetation Restoration Potential Index

Vegetation recovery potential epitomizes the pinnacle condition for optimal vegetation growth attainment [63]. Additionally, due consideration should be afforded to the disparity between the current state of vegetation growth and the optimal growth condition. To encapsulate this differential aspect, this paper computes the vegetation restoration potential index as per Equation (4). To mitigate statistical bias, the 90th percentile value of vegetation cover was adopted as the maximum recoverable cover (VCm). By utilizing the prevailing vegetation cover (VC, %) and the maximum recoverable cover, the vegetation recovery potential index (IVCP, 0–1) was appraised. A value of IVCP ranging from 0 to 1 signifies varying degrees of capacity for prospective vegetation restoration, with 0 signifying the absence of such potential.

(5) $$Ivcp=\left(VC_{m}-VC\right)/VC_{m}$$

where Ivcp was the index of vegetation restoration potential, ${VC}_{m}$ was the maximum cover, and VC was the current vegetation cover.

#### 4.3.4. Mann–Kendall Trend Test

The Mann–Kendall test, as a non-parametric method, has the advantage of being able to test for both linear and non-linear trends and has been widely used in the analysis of time series in hydrology, drought studies, and other fields. Research has found that the MK test trend exhibits autocorrelation, particularly when dealing with long-term time series data, which can affect the scientific accuracy of the results. To correctly eliminate the influence of sequence autocorrelation on the calculation results, the MK test has been improved and modified, resulting in the modified Mann–Kendall (MMK) test. Based on this, this paper will use the MMK trend test to perform a significance test on the spatial changes in vegetation in the northwest region during different periods. The calculation steps for the statistical value S and the standardized test statistic Z are as follows:

For a given time series $X=\left\{x_{1},x_{2},\cdots ,x_{n}\right\}$,

(6) $$S=\sum_{i<j}a_{ij}=\sum_{i<j}\mathrm{sgn}\left(x_{j}-x_{i}\right)$$

(7) $$var(S)=\eta \times \frac{n(n-1)(2n+5)}{18}$$

(8) $$\eta =1+\frac{1}{n(n-1)(n-2)}\times \sum_{i=1}^{n-1}(n-i)(n-i-1)(n-i-2)r_{i}$$

In the formula, S is the test statistic, sgn is the sign function, X is the time series, and $r_{i}$ is the autocorrelation coefficient. Given a significance level of α, if the autocorrelation is significant, the variance, $var\left(S\right),$ is calculated using the modified formula. Finally, the standardized test statistic Z in the MMK trend test can be obtained using the following formula:

(9) $$Z=\left\{\begin{array}{cc} \frac{S-1}{\sqrt{var(S)}} & S>0\\ 0 & S=0\\ \frac{S+1}{\sqrt{var(S)}} & S<0 \end{array}\right.$$

In the formula, Z represents the trend of changes in the time series data. If Z is positive, it indicates an increasing trend in vegetation over time in the study area; conversely, if Z is negative, it indicates a decreasing trend over time. According to the standard normal distribution table, if the absolute value of Z is greater than or equal to 1.64, 1.96, and 2.58, it indicates that the time series has passed the significance tests at the 90%, 95%, and 99% confidence levels, respectively. This corresponds to a slightly significant change, a significant change, and a highly significant change in vegetation. For an overall increasing or decreasing trend, this study selects a significance level of α = 5%, corresponding to a Z value of 1.96.

The NDVI, the LPJ dynamic global vegetation model, and the Mann–Kendall test method demonstrate significant advantages in this study. The NDVI is a commonly used remote sensing index for assessing vegetation coverage and health, providing a direct and intuitive measure of vegetation growth. The NDVI data, derived from remote sensing imagery, offer large-scale spatial and temporal distribution information, making them particularly suitable for studying northwest China, a region characterized by vast areas and diverse ecosystems. Additionally, its long time series supports the analysis of vegetation dynamics and restoration potential. The LPJ model, as a dynamic global vegetation model, simulates interactions between vegetation and climate, including carbon, water, and energy cycles. It compensates for gaps in remote sensing data across time and space, enabling precise predictions of vegetation dynamics under various climatic conditions. This is particularly beneficial for assessing the impacts of climate warming and humidification on vegetation restoration potential. The Mann–Kendall test, as a non-parametric statistical method, is highly robust and insensitive to outliers, making it well suited for analyzing vegetation coverage trends over time. It effectively identifies significant trends in NDVI data, revealing spatiotemporal patterns of vegetation change. Together, these three methods complement each other, providing a solid technical foundation for a comprehensive investigation of vegetation dynamics and restoration potential in northwest China.

## 5. Conclusions

In this study, we selected the normalized vegetation index based on remote sensing images from the AVHRR from 1982 to 2019 in northwest China; explored the spatial and temporal variation characteristics of vegetation in the study area; and predicted the potential for vegetation restoration in northwest China using the similar habitat method. The main conclusions were as follows:

(1) The vegetation cover in northwest China has shown a significant increase from 1982 to 2019, with notable concentrations in northeastern Inner Mongolia, northwestern Xinjiang, eastern and southern Qinghai, and southern Gansu. However, vast areas in central northwest China remain bare or covered with desert soil. The spread of vegetation into surrounding areas, attributed to increased precipitation resulting from the warming and humidification trend in northwest China, has contributed to an expansion of vegetation cover in the region.
(2) The peak of vegetation cover in northwest China is significantly affected by climate warming and the trend of increasing humidity, and the vegetation recovery potential in northwest China is decreasing due to climate warming and increasing humidity. The overall vegetation recovery potential in northwest China is still limited, and the average value of the vegetation recovery potential index of northwest China obtained from the remote sensing data is 0.31, and there is still a 2.3% room for improvement in vegetation cover in this region.
(3) The average value of the vegetation recovery potential index in northwest China obtained from the LPJ model data is 0.14, and there is a 1.1% room for improvement in vegetation cover in this region. The potential for vegetation recovery is higher in central and western Inner Mongolia, northwestern Qinghai, and parts of Xinjiang, while other areas, especially in the east and south, have less potential for vegetation recovery because the level of vegetation cover is already high.

In summary, these research results will provide a theoretical basis for vegetation restoration, desertification control, and land management planning in northwest China, offering valuable insights for future climate adaptation strategies and policy formulation.

## Figures and Tables

**Table 1:** Area proportions for NDVI trend in the northwest (%).

Year	Degraded Significantly	Degraded	Degraded Slightly	No Change	Restored Slightly	Restored	Restored Significantly
1982–2019	21.3	4.89	2.09	29.02	2.8	6.08	33.82
1982–2000	0.1	0.35	0.56	78.36	7.29	7.28	6.06
2000–2019	11.92	7.18	5.03	59.69	6.91	4.2	5.07

**Table 2:** Area proportions for NDVI trend in the northwest (%) (LPJ simulation).

Year	Degraded Significantly	Degraded	Degraded Slightly	No Change	Restored Slightly	Restored	Restored Significantly
1982–2019	0.2	0.75	0.65	37.34	3.04	5.63	52.39
1982–2000	0.93	2.12	1.74	54.4	7.11	8.84	24.86
2000–2019	0.23	0.24	0.15	36.66	7.1	16.76	38.86

**Table 3:** Area proportions for FVC trend in northwest China (%).

	Very Low	Low	Lower	Medium–Low	Medium	Medium–High	High
FVC	<0.05	0.05–0.1	0.1–0.2	0.2–0.4	0.4–0.6	0.6–0.8	>0.8
1982	54.28	4.63	3.11	12.26	9.74	7.63	8.35
2000	52.12	4.24	3.1	11.53	11.62	8.46	8.93
2019	50.63	3.51	2.62	11.35	11.3	10.31	10.28

**Table 4:** Proportion of area (%) with vegetation restoration potential index for northwest China.

	0–0.05	0.05–0.1	0.1–0.2	0.2–0.4	0.4–0.6	0.6–0.8	0.8–1
IVSP (satellite-observed)	40.47%	10.48%	10.94%	8.71%	4.02%	2.79%	22.59%
IVCP (LPJ)	68.97%	1.81%	2.48%	9.99%	7.33%	4.89%	4.53%

**Table 5:** Correlation and significance test between NDVI/FVC and SPEI 12 for northwest China (unit: %).

Indicator	Correlation	Extremely Significant Positive	Significant Positive	Not Positive	Not Negative	Significant Negative	Extremely Significant Negative
NDVI and SPEI 12	Proportion	6.62%	7.27%	45.31%	35.77%	3.83%	1.2%
FVC and SPEI 12	Proportion	6.51%	8.68%	50.19%	31.30%	2.35%	0.97%

**Table 6:** List of drought index division.

Degree	SPEI Value	Class
1	SPEI > −0.5	Near normal
2	−1 < SPEI ≤ −0.5	Mildly dry
3	−1.5 < SPEI ≤ −1	Moderately dry
4	−2 < SPEI ≤ −1.5	Severely dry
5	SPEI ≤ −2	Extremely dry (drought)

## Data Availability

Data are contained within the article.
